# Emodin and rhein decrease levels of hypoxia-inducible factor-1α in human pancreatic cancer cells and attenuate cancer cachexia in athymic mice carrying these cells

**DOI:** 10.18632/oncotarget.21330

**Published:** 2017-09-27

**Authors:** Lijuan Hu, Rui Cui, Hongyi Liu, Feng Wang

**Affiliations:** ^1^ The Graduate School, Tianjin Medical University, Tianjin, China; ^2^ The Institute of Integrative Medicine for Acute Abdominal Diseases, Nankai Hospital, Tianjin, China

**Keywords:** pancreatic cancer, hypoxia-inducible factor-1α, emodin, rhein, cachexia

## Abstract

The transcription factor hypoxia-inducible factor-1 (HIF-1) consists of oxygen-sensitive HIF-1α and constitutive HIF-1β. HIF-1α is undetectable in normal cells, but cancer cells frequently express HIF-1α to support their growth, angiogenesis, and high glycolysis (also known as the Warburg effect). The Warburg effect in cancer cells increases energy expenditure and thus participates in cancer-induced metabolic disorder, cancer cachexia. In the present study, we investigated whether two components of Rheum palmatum, emodin and rhein, inhibited HIF-1α expression in human pancreatic cancer cells and whether the inhibiting effect, if any, attenuated cancer cachexia. Using Western blotting, we demonstrated that emodin and rhein decreased HIF-1α expression in MiaPaCa2 and four other human pancreatic cancer cell lines. We also examined HIF-1α expression when MiaPaCa2 cells were exposed to PX-478, noscapine, and phenethyl isothiocyanate, as these compounds were known to inhibit HIF-1α expression in different cancer cells. PX-478 and noscapine inhibited HIF-1α expression to a less extent than emodin and rhein, and phenethyl isothiocyanate did not inhibit HIF-1α expression in tested concentrations. We obtained evidence that emodin and rhein decreased HIF-1α by decreasing its biosynthesis but not gene transcription or protein stability. When MiaPaCa2 cells were implanted in athymic mice, emodin and rhein inhibited cancer-cell growth and HIF-1α expression. In these athymic mice, emodin and rhein also attenuated two pathological constituents of cancer cachexia, namely high hepatic gluconeogenesis and skeletal-muscle proteolysis. In conclusion, emodin and rhein decrease pancreatic cancer cell's growth and HIF-1α expression and attenuate cancer cachexia in the athymic mice carrying the cancer cells.

## INTRODUCTION

Normal mammalian cells produce energy primarily by oxidative phosphorylation, whereas cancer cells do it mainly by glycolysis even though glycolysis is of low efficiency in energy production. To produce enough energy, cancer cells over-express glucose transporters and glycolytic enzymes to raise glycolysis to high levels. In 1920s, Otto Warburg first described the high glycolysis in cancer cells, so the phenomenon is also called the Warburg effect [1]. How cancer cells manage to over-express glucose transporters and glycolytic enzymes had been unknown, until a transcription factor namely hypoxia-inducible factor-1 (HIF-1) was discovered in 1992 [2].

HIF-1 consists of oxygen-sensitive HIF-1α and constitutive HIF-1β. After HIF-1α is synthesized, its P_402_ and P_564_ residues are hydroxylated by oxygen and prolyl hydroxylase domain proteins-1, -2, and -3 (PHDs-1, -2, and -3). Subsequently, hydroxylated HIF-1α is associated with the Von Hippel-Lindau protein, tagged with ubiquitin, and degraded in proteasomes [3, 4]. Thus, normal mammalian cells have HIF-1β but not HIF-1α. When cells are stressed by hypoxia, HIF-1α is saved from degradation and associated with HIF-1β. The resulting HIF-1 transcriptionally up-regulates its target genes the proteins encoded by which include glucose transporters, glycolytic enzymes, and growth- and angiogenic factors. Thus, when hypoxic cells express HIF-1α, their glycolysis, viability, and angiogenesis increase, so they are more resistant to hypoxic stresses [5].

Cancer cells frequently express HIF-1α and thereby acquire increased glycolysis, viability, and angiogenesis [5, 6]. The mechanisms of cancer-induced HIF-1α are multifactorial, including a decrease in HIF-1α degradation that is associated with intra-tumor hypoxia and an increase in HIF-1α production that is associated with oncogene expression [7, 8]. If HIF-1α expression is inhibited in cancer cells, aggressive behaviors decrease in these cells.

Cancer cachexia is a cancer-induced metabolic syndrome whose pathological constituents include increased energy expenditure, augmented hepatic gluconeogenesis, uncontrolled skeletal-muscle wasting (proteolysis), and unrestrained fat lipolysis [9, 10]. When these pathologies persist, body weight decreases. It remains unclear how these pathologies are initiated, so cancer cachexia is still treated with palliative measures.

Due to the Warburg effect, cancer cells consume a good deal of blood glucose and release lactate as the waste. As a result, hepatic gluconeogenesis increases to recycle tumor-produced lactate to glucose. When the glucose is released to the blood, cancer cells may take it up for glycolysis again. In cancer patients, the glucose-lactate shuttle cannot meet the demand for glucose. Thus, skeletal muscle and adipose tissues undergo catabolic metabolisms to release more glucose precursors for the liver. Consequently, body weight decreases. In this light, the Warburg effect in cancer cells is a triggering event in the pathogenesis of cancer cachexia. Thus, if the Warburg effect decreases following an inhibition of cancer-induced HIF-1α, cancer cachexia may be reversed at least to some extent.

Numerous compounds are reported to inhibit HIF-1α expression, but no HIF-1α inhibitors are clinically available as anti-cancer drugs [11–16]. We undertook the present study to investigate whether emodin and rhein from *Rheum palmatum* inhibited HIF-1α expression in human pancreatic cancer cells. We also investigated whether the HIF-1α-inhibiting effect of emodin and rhein, if any, attenuated cancer cachexia in the athymic mice that carried the cancer cells.

## RESULTS

### The effects of emodin and rhein on HIF-1α in pancreatic cancer cells

**Figure 1:**
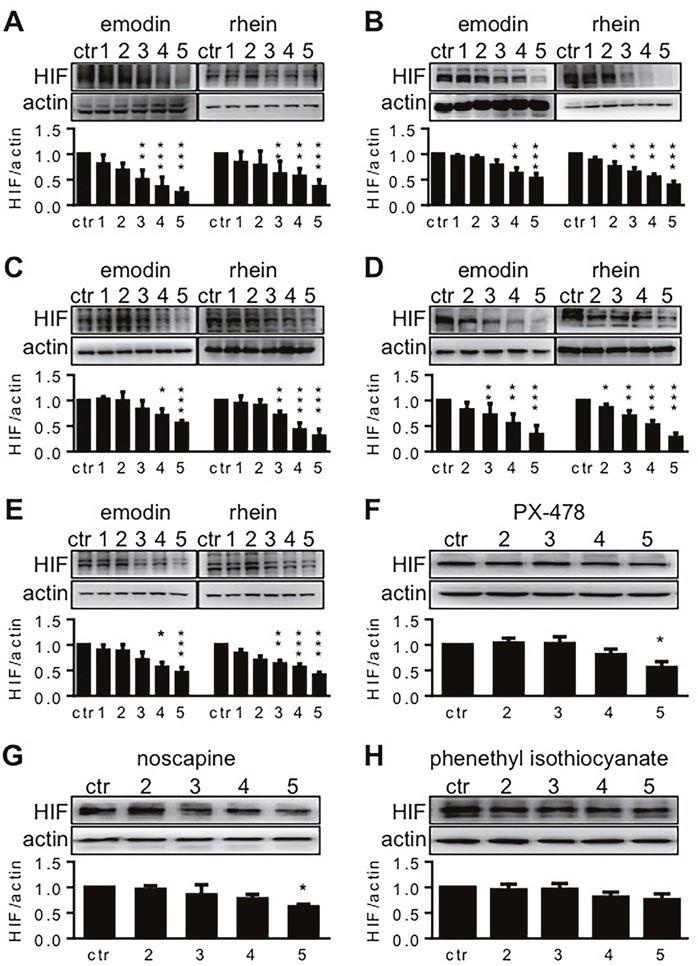
The effects of test reagents on HIF-1α expression Test reagents were diluted in culture media to give the final concentrations of 10, 20, 50, 100, and 200 μM. These concentrations are denoted by 1, 2, 3, 4 and 5, respectively, in this figure. Pancreatic cancer cells were incubated in hypoxia for 6h, using the reagent-containing media. Control cells (ctr.) were incubated without tested reagents. HIF-1α was determined by Western blot, using β-actin for loading control. The upper blots in each panel are representative results for 5-7 experiments, and the lower histograms summarize HIF-1α-actin ratios in all the experiments. ^*^ P<0.05, ^**^ P<0.01, and ^***^ P<0.001, compared to control values. **(A-E)**. The effects of emodin and rhein on HIF-1α expression in AsPC-1 (A), BxPC-3 (B), HPAF-2 (C), MiaPaCa2 (D), and Panc-1 (E) pancreatic cancer cells. **(F-H)**. The effects of PX-478 (F), noscapine (G), and phenethyl isothiocyanate (H) on HIF-1α expression in MiaPaCa2 cells.

When five human pancreatic cancer cell lines were exposed to emodin or rhein, HIF-1α was decreased dose-dependently by either reagent (Figure 1A-1E). In these cell lines, the lowest effective doses of emodin were varied from 50 μM to 100 μM and those of rhein from 20 μM to 50 μM. When five cell lines were exposed to 200μM emodin, their HIF-1α expression levels equaled to 24%-55% of the control value (Figure 1A-1E). When five cell lines were exposed to 200μM rhein, their HIF-1α expression levels equaled to 28%-50% of the control value (Figure 1A-1E). These results demonstrate that both emodin and rhein inhibited HIF-1α expression in pancreatic cancer cells.

PX-478, noscapine, and phenethyl isothiocyanate (PEICT) inhibited HIF-1α expression in different cancer cells [14–16]. In the present study, we used them as references to assess the effects of emodin and rhein on HIF-1α expression in pancreatic cancer cells. To this end, these drugs were administered to MiaPaCa2 cells in the same concentrations as when emodin and rhein were used (20 μM-200 μM). HIF-1α expression was significantly decreased only when PX-478 and noscapine were used in 200 μM (Figure 1F and 1G). When MiaPaCa2 cells were exposed to the highest concentration of PX-478 and noscapine, HIF-1α expression levels equaled to 55% and 62% of the control value, respectively. PEICT did not change HIF-1α expression in MiaPaCa2 cells (Figure 1H). Taken together, these results demonstrate that emodin and rhein inhibited HIF-1α expression effectively in the cells examined.

### The molecular biology of HIF-1α expression when MiaPaCa2 cells were treated with emodin and rhein

**Figure 2:**
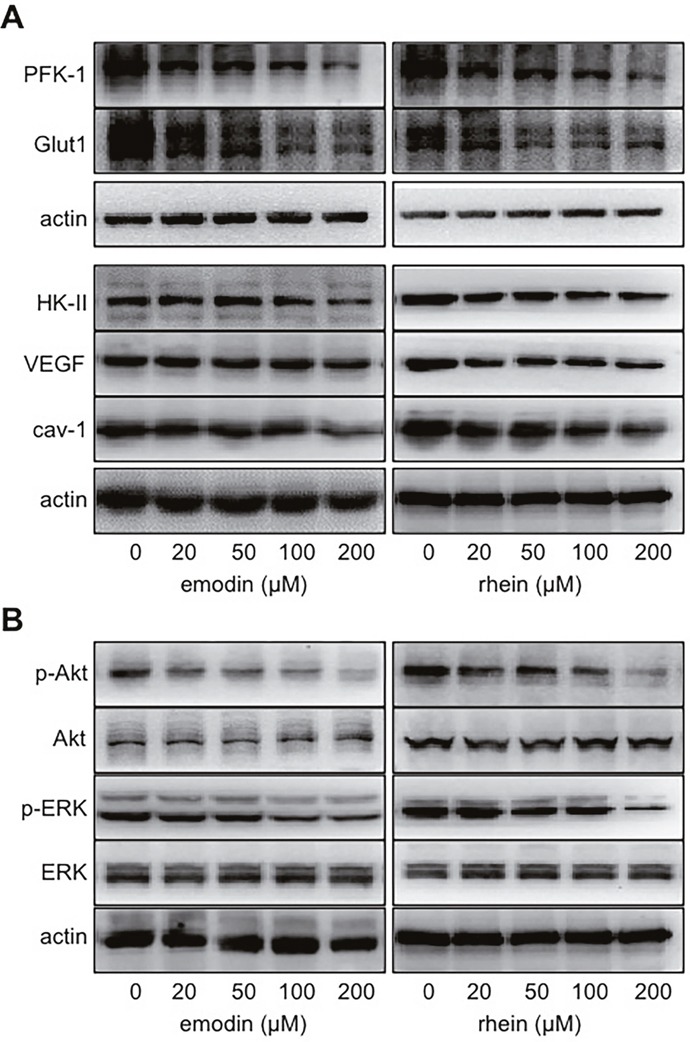
The effects of emodin and rhein on HIF-1α-related proteins MiaPaCa2 cells were incubated in hypoxia for 6h, using culture media containing emodin and rhein as indicated. Western blots were performed, using β-actin for loading control. **(A)**. Phosphofructokinase-1 (PFK-1), glucose transporter-1 (Glut1), hexokinase-2 (HK-II), vascular endothelial growth factor (VEGF), and caveolin-1 (cav-1) were determined. **(B)**. Akt and extracellular signal-regulated kinase 1/2 (ERK) were determined in total and in phosphorylatedforms (p-).

When HIF-1α decreases, so do the proteins whose expression are regulated by HIF-1α. In MiaPaCa2 cells, we determined glucose transporter-1 (Glut1), hexokinase-2 (HK-II), phosphofructokinase-1 (PFK-1), vascular endothelial growth factor (VEGF), and caveolin-1 (cav-1) as such proteins. These proteins were decreased unanimously in the presence of emodin or rhein (Figure 2A). This result is expected to be secondary to the inhibition of HIF-1α expression in the same cells.

Two signaling pathways, which include Akt and extracellular signal-regulated kinase 1/2 (ERK1/2) respectively, induce HIF-1α expression by increasing HIF-1α biosynthesis [8, 11, 15, 17–20]. The proximal ends of these pathways start from growth-factor receptors on cellular surface. After these receptors are activated, the signal is passed forward when kinases in the signaling pathways are phosphorylated one after another [8]. Emodin and rhein decreased phosphorylated Akt and ERK/1/2 (p-Akt and p-ERK1/2) without changing total Akt and ERK1/2 (Figure 2B). This result suggests that emodin and rhein decrease levels of HIF-1α by down-regulating the intracellular signaling pathways that lead to the expression of HIF-1α.

**Figure 3:**
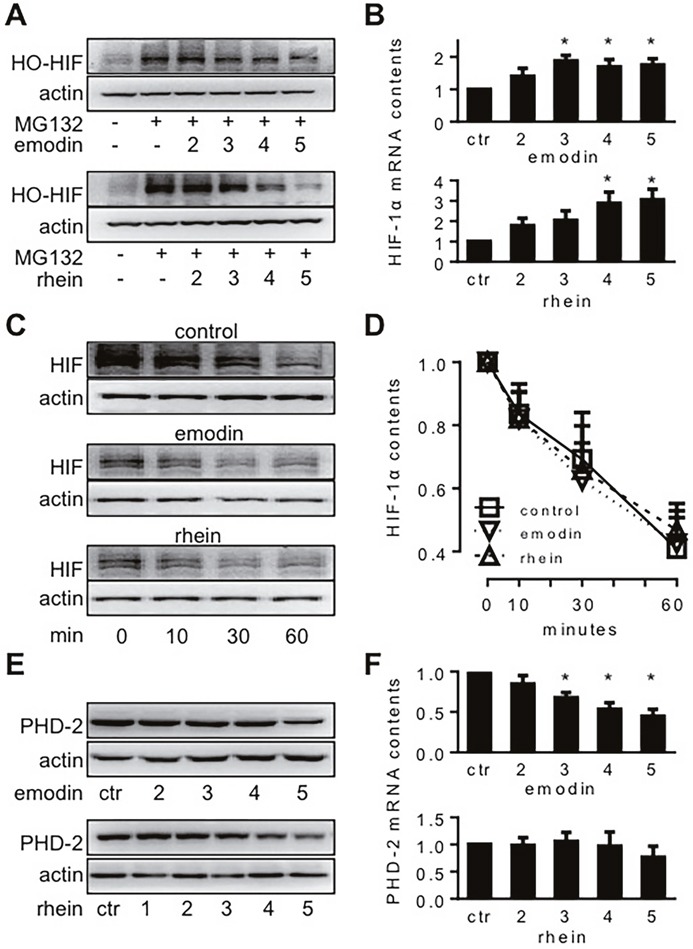
The molecular biology of HIF-1α expression in MiaPaCa2 cells treated with emodin or rhein MiaPaCa2 cells were treated with emodin or rhein for 6h, using untreated cells for control (ctr). Unless indicated otherwise, emodin and rhein were used in 10, 20, 50, 100, and 200 μM, and these concentrations are denoted in this figure by 1, 2, 3, 4 and 5, respectively. **(A)**. Cells were incubated in normoxia for 6h. Culture media were supplemented with MG132 (20 μM) to save hydroxylated HIF-1α (HO-HIF-1α) from degradation. HO-HIF-1α was determined by Western blot using an antibody for P_564_ HO-HIF-1α. **(B)**. After 6h hypoxic incubation, HIF-1α mRNA was determined by real-time RT-PCR; n=6, ^*^P<0.05 compared to control value. **(C** & **D)**. HIF-1α degradation test: MiaPaCa2 cells first underwent 6h hypoxic incubation to accumulate HIF-1α, using normal media (control) or media containing emodin (100 μM) or rhein (100 μM). After the incubation, CHX was added to all media (100 μg/ml) to stop protein biosynthesis. Cells were further incubated for 0, 10, 30, and 60 min to study the ways HIF-1α was decreased during this period. (C). Representative results. (D). Cumulative results, n=5. **(E** & **F)**. After 6h hypoxic incubation, PHD-2 and its mRNA were determined by Western blot (E) and real-time RT-PCR (F), respectively. ^*^P<0.05, compared to control value, n=6.

When mammalian cells are in normoxia, HIF-1α is hydroxylated by PHDs and O_2_ before it is degraded in proteasomes [3, 4]. If proteasome activity is inhibited by MG-132, hydroxylated HIF-1α is stable even in normoxia [21]. In the present study, emodin and rhein decreased hydroxylated HIF-1α in normoxic MiaPaCa2 cells in the presence of MG-132 (Figure 3A). Thus, emodin and rhein decreased HIF-1α biosynthesis in both hypoxia and normoxia. HIF-1α mRNA increased when MiaPaCa2 cells were exposed to emodin and rhein (Figure 3B). It may reflect a feedback when the protein of HIF-1α was decreased by emodin and rhein.

Next, we investigated whether emodin and rhein decreased HIF-1α stability. When cells are in hypoxia, their HIF-1α degradation may not be inhibited totally. If HIF-1α production is inhibited in hypoxic cells, their HIF-1α contents will decrease overtime as in normoxic cells [21]. To study HIF-1α degradation in hypoxic conditions, we incubated MiaPaCa2 cells in hypoxia for 6h without or with emodin (200 μM) or rhein (200 μM). Then, CHX was added to culture media (100 μg/ml) to inhibit HIF-1α production, and the cells were further incubated in hypoxia for 10, 30, and 60 minutes. By examining HIF-1α contents at these time points, we appreciated the way HIF-1α was degraded in the absence or presence of emodin and rhein. After CHX was added, the contents of HIF-1α were decreased gradually in control cells (Figure 3C). As expected, HIF-1α expression was decreased by emodin or rhein before the start (Min-0) of the degrading period (Figure 3C). After CHX was added, however, the way HIF-1α was degraded in the emodin- or rhein-treated cells was similar to that seen in the control cells (Figure 3C and 3D). The data suggest that the inhibition of HIF-1α expression induced by emodin and rhein was not a result of increased HIF-1α degradation. In separate experiments, we determined PHD-2 protein and mRNA in the presence and absence of emodin and rhein. Emodin decreased PHD-2 expression at both protein and mRNA levels, and rhein decreased PHD-2 protein (Figure 3E and 3F). These data may reflect a feedback regulation in PHD-2 expression when HIF-1α, as a substrate of PHD-2, was decreased by emodin and rhein.

### The effects of emodin and rhein on the viability of MiaPaCa2 cells *in vivo*

**Figure 4:**
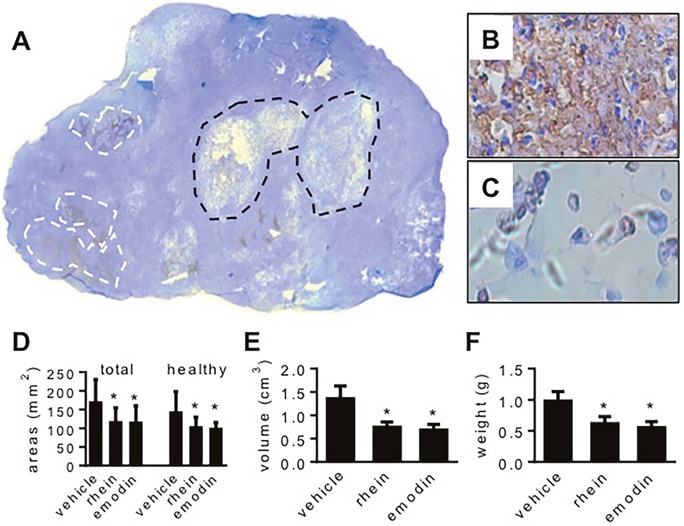
The effects of emodin and rhein on the viability of MiaPaCa2 cells *in vivo* MiaPaCa2 cells grew as subcutaneous tumors in three groups of athymic mice (10 mice per group) for 8 weeks. In the last 4 weeks, the mice were treated with emodin, rhein, or vehicle, respectively. **(A-C)**. A tumor whose host was treated with vehicle is used to show common histological features. (A). In the whole-section image (original magnification: 50x), white lines surround typical apoptotic regions and black lines surround central necrosis. (B). A region full of apoptosis (original magnification: 400x). (C). Central necrosis (original magnification: 400x). **(D-F)**. Data shown in these panels are derived from all tumors. (D). Total section area and the section area that was occupied by healthy tissue. (E). Tumor volume. (F). Tumor weight. ^*^ P<0.05.

**Figure 5:**
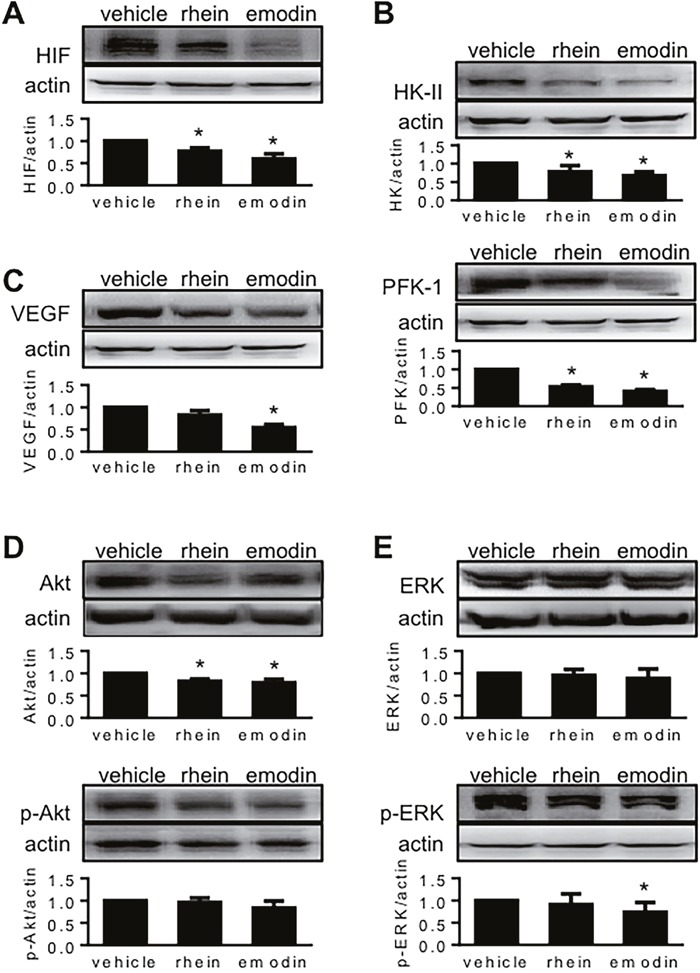
The effects of emodin and rhein on HIF-1α-related proteins in MiaPaCa2 cells *in vivo* See the legend of Figure 4 for study design. HIF-1α and related proteins were determined in tumor grafts by Western blot, using β-actin as loading control. In these panels, the upper blots are representative results, and the lower histograms show target-control ratios in all tumors. The proteins examined included HIF-1α **(A)**, HK-II **(B)**, PFK-1 (B), VEGF **(C)**, Akt **(D)**, p-Akt (D), ERK1/2 **(E)**, and p-ERK1/2 (E). ^*^P<0.05, compared to the tumors whose carriers were treated with vehicle.

All mice survived the experiment. When tumor histology was examined, several features were seen independent of whether the tumor carriers were treated with emodin, rhein or vehicle. Figure 4A-4C show these features using a tumor whose carrier was treated with vehicle. Following transferase-mediated deoxyuridine triphosphate-biotin nick end labeling (TUNEL) staining, the section regions that were full of apoptotic cells were stained in brown (Figure 4A and 4B). Chronic central necrosis was shown as a hollow region (Figure 4A) wherein dead cells were scattered sporadically (Figure 4C). When we measured total section area and the section area that was occupied by healthy tumor tissue, we found that these areas decreased when tumor carriers were treated with emodin or rhein (Figure 4D). When dead-cell areas (i.e. apoptosis + central necrosis) were quantified, no significant difference was seen in three groups of tumors (data not shown). When dead-cell areas were calculated in percent of total section, no significant difference was found either (vehicle-treated: 15.5% ± 2.5%, emodin-treated: 16.0% ± 3.2%, rhein-treated 16.1% ± 4.8%). However, emodin and rhein treatments decreased tumor weight and volume (Figure 4E and 4F). Emodin and rhein also decreased the expression of HIF-1α, HK-II and PFK-1 in tumor grafts (Figure 5A and 5B). However, the expression of VEGF was significantly decreased by emodin but not rhein (Figure 5C). In the same tumor grafts, emodin and rhein decreased total Akt but not p-Akt (Figure 5D). Neither emodin nor rhein changed total ERK1/2, but emodin decreased p-ERK1/2 (Figure 5E). These indicate that, *in vivo*, emodin and rhein had different effects on the two signaling pathways for HIF-1α expression.

### The effects of emodin and rhein on tumor-carrier's energy homeostasis

**Figure 6:**
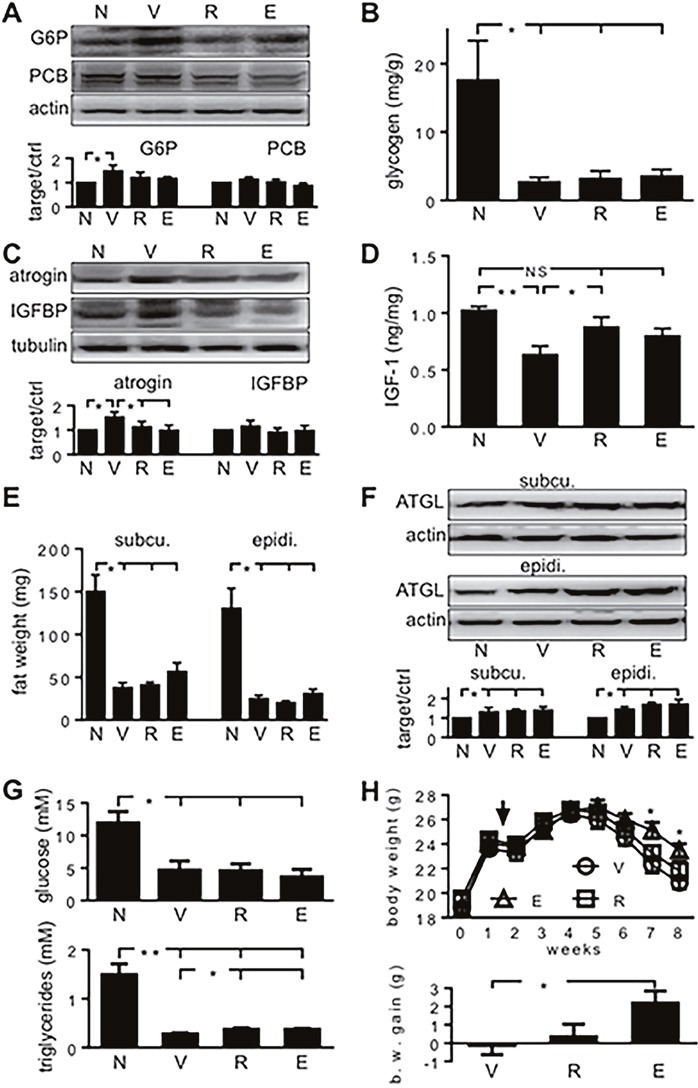
The effects of emodin and rhein on energy homeostasis of tumor-carrying athymic mice MiaPaCa2 cells grew subcutaneously in athymic mice for 8 weeks. In the last 4 weeks, the mice were treated with emodin (E, n=10), rhein (R, n=10), or vehicle (V, n=10), respectively. A group of normal athymic mice (N, n=5) were also included. When Western-blotting data are shown in A, C and F, the upper parts are representative blotting, and the lower histograms show data for all mice. **(A)**. Hepatic glucose-6-phosphatase (G6P) and pyruvate carboxylase (PCB) were determined by Western blot. **(B)**. Glycogen contents were determined in the liver. **(C).** Atrogin-1 and insulin-like growth factor binding protein-3 (IGFBP) were determined in skeletal muscle by Western blots. **(D)**. IGF-1 was determined in skeletal muscle, and the results were normalized with the weight of samples. **(E)**. Subcutaneous and epididymal fat pads were weighed. **(F)**. Adipose triglyceride lipase (ATGL) was determined in subcutaneous and epididymal fat by Western blot. **(G)**. Plasma glucose and triglycerides were determined. **(H)**. The upper part: Body weight in three groups of tumor carriers. The arrow indicates when cancer cells were implanted. The lower part: Net difference in body weight over 8 weeks. ^*^P<0.05 and ^**^P<0.01; NS, not significant.

Hepatic gluconeogenesis starts when pyruvate carboxylase (PCB) carboxylates pyruvate and ends when glucose-6-phosphatase (G-6-Pase) hydrolyzes glucose-6-phosphate to give glucose. In the present study, hepatic G-6-Pase increased when tumor-carrying mice were only treated with vehicle (Figure 6A). It indicates that hepatic gluconeogenesis increased when mice carried MiaPaCa2 cells. When tumor carriers were treated with emodin or rhein, hepatic G-6-Pase expression was normal. The data suggest that cancer-induced hepatic gluconeogenesis was attenuated when HIF-1α expression in cancer cells was decreased by emodin and rhein. Hepatic PCB expression tended to increase in tumor carriers, but the change was not significant statistically (Figure 6A). All tumor-carrying groups showed significant decrease in hepatic glycogen, compared to the control value, and no significant difference was found between any tumor-carrying groups (Figure 6B). Thus, cancer-induced decrease in hepatic glycogen cannot be relieved by emodin and rhein.

In skeletal muscle, protein degradation is regulated by factors such as atrogin-1, and protein production is regulated by factors such as insulin-like growth factor-1 (IGF-1) [22–24]. Further, the amount of free (active) IGF-1 is regulated by IGF binding proteins (IGFBPs). In the present study, atrogin-1 expression was increased in vehicle-treated tumor carriers but not in those that were treated with emodin and rhein (Figure 6C). Skeletal-muscle IGFBP3 expression was similar in the different mice (Figure 6C). Skeletal-muscle IGF-1 was decreased in vehicle-treated tumor carriers, as compared to control value (Figure 6D). When tumor carriers were treated with emodin or rhein, skeletal-muscle IGF-1 was normal (Figure 6D). Taken together, these results indicate that cancer-induced skeletal-muscle wasting was attenuated when HIF-1α expression in cancer cells was inhibited by emodin or rhein.

The weight of subcutaneous and epididymal fat pads was decreased in all tumor carriers (Figure 6E). This suggests that the tumor carriage increased lipolysis. Adipose triglyceride lipase (ATGL) is a key regulator in cancer-induced lipolysis [25]. In the present study, tumor carriers showed increased ATGL expression in subcutaneous and epididymal fat (Figure 6F). No significant differences were found when ATGL expression was compared in the different groups of tumor carriers (Figure 6F). Plasma glucose and triglycerides were decreased in all tumor carriers (Figure 6G). Emodin and rhein had no effects on plasma glucose but they attenuated the cancer-induced decrease in triglycerides (Figure 6G). The effect of tumor burden on plasma glucose levels was comparable to the effect of tumor burden on hepatic glycogen contents. Further, the result of plasma glucose demonstrates once more that glucose turnover was increased when athymic mice carried pancreatic cancer cells.

Previously, we showed that when growing athymic mice carried MiaPaCa2 cells for 15 weeks, their body-weight gain was less than normal value [26]. In the present study, when tumor-carrying athymic mice were treated with emodin, their body weight was significantly increased, compared to that seen in vehicle-treated tumor carriers (Figure 6H, the upper part). Body weight in the rhein-treated mice tended to increase, but the change was not significant statistically (Figure 6H, the upper part). When body weight was examined as net difference over experiment, a significant improvement was seen in the emodin-treated mice and an insignificant improvement was seen in the rhein-treated ones (Figure 6H, the lower part). These data suggest that emodin and rhein treatment may improve cancer-induced decrease in body weight.

## DISCUSSION

Emodin and rhein are components of *Rheum palmatum* that is also known as Chinese rhubarb. By chemistry, emodin and rhein are 1,3,8-trihydroxy-6-methylanthracene-9,10-dione and 4,5-dihydroxy-9,10-dioxoanthracene-2-carboxylic acid, respectively. Previous studies have shown that emodin and rhein combat pathologies such as inflammation, cancer, and obesity-associated metabolic disorders [27–34], but molecular mechanisms underlying these therapeutic effects are yet to clarify. Recently, we demonstrated that emodin inhibited hepatic HIF-1α expression in the mice that had diet-induced obesity and also showed evidence that the HIF-1α-inhibiting effect of emodin was a result of decreased HIF-1α biosynthesis [21]. In the present study, emodin and rhein inhibited HIF-1α expression in MiaPaCa2 and four other pancreatic cancer cell lines *in vitro* and also in MiaPaCa2 cells *in vivo* when the cells grew as subcutaneous tumors in athymic mice. In addition, we demonstrated evidence that suggests that the HIF-1α-inhibiting effect of emodin and rhein in MiaPaCa2 cells also resulted from a decrease in HIF-1α biosynthesis. When three compounds that were known as HIF-1α inhibitors were tested in MiaPaCa2 cells for HIF-1α inhibition, two of them were less effective than emodin and rhein, with the last one being unable to inhibit HIF-1α expression. This being the case, emodin and rhein appear to be potent HIF-1α inhibitors for pancreatic cancer cells.

Two signaling pathways, which include Akt and ERK1/2 respectively, induce HIF-1α expression [8, 17–20]. When these pathways are activated, the kinases that constitute these pathways are phosphorylated one after another. As a result, translation-regulating proteins such as 4E-BP1, p70 S6 kinase, and eIF-4E are activated, so that HIF-1α biosynthesis is increased. In the present study, emodin and rhein decreased p-Akt and p-ERK1/2 *in vitro*. In tumor grafts, however, the emodin- and rhein-induced decrease in HIF-1α expression was associated with a decrease in total Akt but not p-Akt. It suggests that long-term emodin or rhein treatment *in vivo* may attenuate the activity of this signaling pathway by decreasing its constituent kinases. Regarding the other signaling pathway involving ERK1/2, emodin treatment decreased p-ERK1/2 but not total ERK1/2, whereas rhein treatment had no effects on either total ERK1/2 or p-ERK1/2. These data suggest that, when emodin and rhein are used chronically *in vivo*, they may have different effects on the two signaling pathways that regulate HIF-1α biosynthesis.

Emodin and rhein decreased tumor volume and weight, which suggests that both drugs inhibited cancer-cell growth. When tumor section was analyzed, emodin and rhein decreased both total section area and the section area that was occupied by healthy tissue. Neither emodin nor rhein changed the section area that was occupied by dead cells. Thus, emodin and rhein may inhibit tumor growth by decreasing cell proliferation rather than promoting cell death. When emodin and rhein decreased HIF-1α in MiaPaCa2 cells *in vitro*, five HIF-1-regulated proteins examined were decreased unanimously, including three regulators of glycolysis, namely Glut1, HK-II, and PFK-1. When emodin and rhein decreased HIF-1α in MiaPaCa2 cells *in vivo*, HK-II and PFK-1 were decreased as well. These results suggest the inhibition of HIF-1α expression induced by emodin and rhein in MiaPaCa2 cells was associated with decreased Warburg effect in the cancer cells. In keeping with this notion, when we silenced the HIF-1α gene by RNA interference, the Warburg effect was decreased in MiaPaCa2 cells [26].

In the present study, MiaPaCa2-cell implantation in athymic mice increased hepatic gluconeogenesis, disrupted skeletal-muscle protein homeostasis, and stimulated fat lipolysis. However, the abnormalities in the liver and skeletal muscle did not appear when the tumor carriers were treated with emodin or rhein. These results are in keeping with the notion that the inhibition of HIF-1α expression by emodin or rhein decreased the Warburg effect in cancer cells and thereby attenuated cancer cachexia. However, emodin and rhein did not correct cancer-induced decreases in hepatic glycogen and in plasma glucose. These suggest that emodin and rhein cannot normalize all cancer-induced changes in glucose homeostasis. Although emodin and rhein attenuated cancer-induced decrease in plasma triglycerides, they had no effects on cancer-induced changes in fat weight and ATGL expression. Recent studies have shown that cancer cells consume a good deal of fatty acids to support their survival [35–38]. In the present study, the cancer-induced lipolysis may result not only from the Warburg effect but also from direct consumption of fatty acids by the cancer cells.

Previously, when we implanted HIF-1α-negative MiaPaCa2 cells and wild-type MiaPaCa2 cells in different athymic mice, significant decrease in body-weight gain was seen in the presence of the wild-type cells but not the HIF-1α-negative ones [26]. In the present study, body weight was increased significantly in emodin-treated tumor carriers, as compared to that seen in vehicle-treated tumor carriers. Although rhein treatment did not improve body weight significantly, it indeed corrected cancer-induced gluconeogenesis and proteolysis.

In conclusion, emodin and rhein inhibited HIF-1α expression in human pancreatic cancer cells both *in vitro* and *in vivo*. The induced inhibition of HIF-1α expression was associated not only with a decrease in cancer cell's growth but also with an improvement in cancer-induced hepatic gluconeogenesis and skeletal-muscle wasting. Emodin and rhein may be used as anticancer drugs that both sabotage malignant tumor cells and improve energy homeostasis in the tumor carrier.

## MATERIALS AND METHODS

### Cell lines and reagents

AsPC-1, BxPC-3, HPAF-2, MiaPaCa2, and Panc-1 human pancreatic cancer cells were purchased from the Cell Bank of Chinese Academy of Science (Shanghai, China). RPMI-1640 and Dulbecco modified Eagle's culture media, fetal bovine serum, and phosphate buffered solution (PBS, pH 7.4) were bought from Thermo Fisher Scientific (Carlsbad, CA). Emodin was bought from SigmaAldrich (St. Louis, MO; #E7881) and Solarbio Life Sciences (Beijing, China; #SE8050). Rhein was bought from SigmaAldrich (#R7269) and Heowns Biochemical (Tianjin, China; #D-52000). The inhibitor of protein synthesis, cycloheximide (CHX, #C7698), and the proteasome inhibitor, MG-132 (#M8699), were purchased from SigmaAldrich.

### Experiments in cell culture

Human pancreatic cancer cells were grown in serum-containing (10%) media at 37°C in normoxia (95% humidified air and 5% CO_2_) and entered experimental incubation when they were 80%-90% confluent. Serum-free media were used for the incubation. Prior to the incubation, test reagents were added to culture media. Unless indicated otherwise, the experimental incubation lasted for 6h in hypoxia. Cells were put in a hypoxic chamber. A mix of N_2_ (95%) and CO_2_ (5%) was flushed into the chamber [21, 26]. When oxygen level decreased to 1%, the chamber was sealed and put at 37°C.

### Implantation of MiaPaC2 cells in athymic mice

Male athymic Balb/c mice were bought from Hua-Fu-Kang Bioscience (Beijing, China). When arriving at our hospital, they were 4-5 weeks old and weighed 17-20 g. We followed the guide for the care and use of laboratory animals, 8th edition (NIH, 2011). After acclimation, mice were designated to three groups (10 mice per group), and 3 × 10^6^ MiaPaCa2 cells were injected subcutaneously in each mouse. Afterwards, all mice were kept for 8 weeks. In the last 4 weeks, emodin and rhein were dissolved in PBS and administered by gavage (50 mg/kg) in two groups, respectively. Vehicle was administered by gavage in the last group. All gavage treatments were given once a day and five days a week. A group of intact athymic mice (n = 5) were also included. When mice were sacrificed, they were anesthetized using 5% chloral hydrate and exsanguinated in the orbital sinus. Blood was collected and centrifuged (1,500 *g*, 10 min) to obtain plasma. Subcutaneous tumor and inguinal fat pads were removed surgically, and so was the skeletal muscle in hind legs. The abdominal cavity was opened. The liver and epididymal fat pads were removed.

### Western blotting assay

We performed Western blots to determine HIF-1α, P_564_ hydroxylated HIF-1α, PHD-2, Glut1, HK-II, PFK-1, VEGF, cav-1, Akt, p-Akt, ERK1/2, p-ERK1/2, PCB, G-6-Pase, atrogin-1, IGFBP-3, and ATGL. β-Actin and β-tubulin were assayed as loading controls. Cell Signaling Technology (Danvers, MA) produced the antibodies for Akt (#2964), PHD-2 (#3293), HO-HIF-1α (#3434), p-ERK (#9101), ERK (#9102), and p-Akt (#9271). Santa Cruz Biotechnology (Santa Cruz, CA) produced the antibodies for HK-II (#6521), G-6-Pase (#27196), PFK-1 (#377346), PCB (#43228), and β-actin (#47778). Abcam (Cambridge, UK) produced the antibodies for Glut1 (#115730), cav-1 (#32577), and ATGL (#3370-1). Novus Biologicals (Littleton, CO), BD Biosciences (Franklin Lakes, NJ), ECM Biosciences (Versailles, KY), R&D Systems (Minneapolis, MN), and Proteintech (Chicago, IL) produced the antibodies for HIF-1α (#100-449), VEGF (#555036), atrogin-1 (#AP2041), IGFBP3 (#MAB305), and β-tubulin (#66240-1), respectively.

Whole-cell proteins were extracted using RIPA lysis buffer. Then, they were separated in polyacrylamide gel, transferred to a piece of polyvinylidene difluoride membrane, and incubated with a primary antibody at 4°C overnight. After incubation with a secondary antibody for 1h, specific blotting was visualized using an enhanced ECL detection kit. Blots were digitalized using the software of ImageJ (version 1.46, NIH). Data of target and control proteins were related to each other, giving the relative values of target proteins. The values in the control group were averaged, and the results were used as the baseline (100% or 1) to which other groups’ data were related.

### Real-time reverse transcription polymerase chain reaction

We performed real-time reverse transcription polymerase chain reaction (RT-PCR) to quantify HIF-1α and PHD-2 genes’ transcripts, using the β-actin gene for control [21]. Total RNAs were extracted, using the Tiangen RNA extraction kit. cDNAs were synthesized, using the Scientific RevertAid first strand cDNA synthesis kit (Thermo #K1622). Three pairs of primers for the HIF-1α, PHD-2, and β-actin genes were 5′-ccgaattgatgggatatgag-3′ & 5′-tggcaagcatcctgtactgt-3′, 5′-tacaaggtacgcaataactg-3′ & 5′-tctttaccgaccgaatctga-3′, and 5′-gatagcacagcctggatagca-3′ & 5′-actgggacgacat ggagaaaa-3′, respectively. The reaction had 40 cycles, with each cycle having 10 seconds at 95°C and 20 seconds at 60°C.

### Histology

Each tumor was cut and embedded in paraffin so that when it was sectioned, the maximum section was obtained. The sections (4-μm thick) were stained using a kit of TUNEL (S7100, Millipore, Temecula, CA) and counter-stained with hematoxylin [38]. In a microscope, tumor morphology was captured region by region, using a built-in camera (DFC500; Leica Camera). Photos were merged to reconstitute whole-section image, using the Leica Application Suite software. Section areas were measured, using the software of ImageJ.

### Other assays

Plasma glucose and triglycerides were determined, using assay kits produced by Rongsheng Life Pharmacological (Shanghai, China) and Jiancheng Bio-engineering (Nanjing, China). Hepatic glycogen was determined, using a kit produced by Jiancheng Bioengineering. IGF-1 was determined in skeletal muscle, using a kit produced by Elabscience Biotechnology (Wuhan, China).

### Statistics

Data are means ± SEM. Analysis of variance was employed to evaluate differences in groups, using Statistical Product and Service Solutions and Graph-Pad Prism. P<0.05 was considered statistically significant.

## Abbreviations

ATGL: adipose triglyceride lipase
cav-1: caveolin-1
CHX: cycloheximide
ERK1/2: extracellular signal-regulated kinase 1/2
G-6-Pase: glucose-6-phosphatase
Glut1: glucose transporter-1
HIF-1: hypoxia-inducible factor-1
HK-II: hexokinase-2
IGF-1: insulin-like growth factor-1
IGFBP3: insulin-like growth factor binding protein-3
PBS: phosphate buffered solution
PCB: pyruvate carboxylase
PEICT: phenethyl isothiocyanate
PFK-1: phosphofructokinase-1
PHD: prolyl hydroxylase domain protein
RT-PCR: reverse transcription polymerase chain reaction
TUNEL: transferase-mediated deoxyuridine triphosphate-biotin nick end labeling
VEGF: vascular endothelial growth factor.
